# Digital Contact Tracing Implementation Among Leaders and Health Care Workers in a Pediatric Hospital During the COVID-19 Pandemic: Qualitative Interview Study

**DOI:** 10.2196/64270

**Published:** 2024-11-05

**Authors:** Brynn O'Dwyer, Mirou Jaana, Charles Hui, Samia Chreim, Jennifer Ellis

**Affiliations:** 1 Telfer School of Management University of Ottawa Ottawa, ON Canada; 2 Division of Infectious Diseases, Immunology, and Allergy Department of Pediatrics Children's Hospital of Eastern Ontario Ottawa, ON Canada; 3 Department of Pediatrics Faculty of Medicine University of Ottawa Ottawa, ON Canada; 4 Kemptville District Hospital Kemptville, ON Canada

**Keywords:** COVID-19, surveillance, technology, digital contact tracing, qualitative, hospitals, Reach, Effectiveness, Adoption, Implementation, and Maintenance framework, RE-AIM

## Abstract

**Background:**

Health systems had to rapidly implement infection control strategies to sustain their workforces during the COVID-19 pandemic. Various outbreak response tools, such as digital contact tracing (DCT), have been developed to monitor exposures and symptoms of health care workers (HCWs). Limited research evidence exists on the experiences with these technologies and the impacts of DCT innovations from the perspective of stakeholders in health care environments.

**Objective:**

This study aims to identify the factors influencing the adoption of DCT, highlight variations in perspectives across 3 key stakeholder groups concerning the impact of DCT, and provide benchmarking evidence for future pandemic preparedness.

**Methods:**

Guided by the Reach, Effectiveness, Adoption, Implementation, and Maintenance (RE-AIM) framework, we conducted an exploratory qualitative study to investigate the implementation and impact of DCT at the Children’s Hospital of Eastern Ontario between December 2022 and April 2023. We conducted 21 semistructured interviews with key stakeholders, including health care administrators (6/21, 29%), occupational health and safety specialists (8/21, 38%), and HCWs (7/21, 33%). Stakeholders were asked about the factors influencing engagement with the DCT tool, organizational-level uptake, the implementation process, long-term use and sustainability of DCT, and unintended consequences. Verbatim transcripts were subject to thematic analysis using NVivo (QSR International).

**Results:**

The implementation of DCT was viable and well received. End users indicated that their engagement with the DCT tool was facilitated by its perceived ease of use and the ability to gain awareness of probable COVID-19 exposures; however, risk assessment consequences and access concerns were reported as barriers (*reach*). Participants commonly agreed that the DCT technology had a positive influence on the hospital’s capacity to meet the demands of COVID-19 (*effectiveness*). Implementors and occupational specialists referred to negative staffing impacts and the loss of nuanced information as unintended consequences (*effectiveness*). Safety-focused communication strategies and having a DCT tool that was human-centered were crucial factors driving staff *adoption* of the technology. Conversely, *adoption* was challenged by the misaligned delivery of the DCT tool with HCWs’ standard practices, alongside the evolving perceived threat of COVID-19. Stakeholders collectively agreed on the viability of DCT and its applicability to infectious disease practices (*maintenance*).

**Conclusions:**

Hospital stakeholders were highly satisfied with DCT technology and it was perceived as feasible, efficient, and having a positive impact on organizational safety. Challenges related to the alignment and delivery of DCT, alongside the evolving perspectives on COVID-19, posed obstacles to continued adoption by HCWs. Our findings contribute to evidence-based practices and present benchmarks that can inform preparedness for future pandemics and infectious disease outbreaks and help other organizations implement similar technologies.

## Introduction

### Background

Contact tracing is a nonpharmaceutical intervention aimed at limiting the transmission of infectious diseases by identifying infected individuals, investigating others who might have come in contact with them, and collecting relevant information to inform the guidance of prevention measures [1-4]. During the SARS-CoV-2 (COVID-19) pandemic, the rising number of infections demonstrated the critical role of contact tracing in managing disease exposures and disrupting the chain of transmissions [5]. As the urgency to respond to the COVID-19 pandemic increased, traditional contact tracing approaches (eg, phone calls and interviews) proved resource intensive and inadequate for managing the scale of infections [6]. As a result, several health systems have implemented and developed various technological solutions to identify, notify, and manage individuals potentially exposed to COVID-19 [6-8].

Given the inherent close contact in health care environments and the infectious nature of communicable diseases like COVID-19, contact tracing has proven effective in safeguarding high-risk patients and health care workers (HCWs), while also lowering hospital-acquired infections [9-13]. While digital contact tracing (DCT) has shown considerable success in community settings [6,14-16], its integration into hospital environments has been limited [17]. Early findings suggest that DCT, through facilitating comprehensive management of COVID-19 cases, enhances hospitals’ capacity to maintain safety, mitigate infection risk, and sustain operations during the pandemic [18-23]. To date, various technologies (eg, closed-circuit television, real-time locating systems, Wi-Fi access point logs, Bluetooth-based systems, electronic medical record [EMR] systems, and web applications) have been used to augment DCT [9,10,18-26]. Nonetheless, the absence of a universally adopted technology [24] indicates that there may be inherent challenges in implementing these technologies in complex health care settings, such as hospitals.

The Canadian health care system faced multi-layered challenges during the pandemic, such as notable increases in hospitalizations, disruptions to health service delivery, and a growing shortage of human health resources (HHRs) [27,28]. Despite the advances in digital surveillance during COVID-19, the need for developing strategies to improve infectious disease prevention, preparedness, and recovery efforts has persisted [29-31]. In Ontario, hospitals are mandated to report probable or confirmed hospital-acquired infections of substantial public health concern to their respective public health authorities [32]. In addition, Public Health Ontario established surveillance programs to detect and track positive cases among patients and staff [33]. Given these requirements and the rise of DCT technologies, it is important to understand how these innovations are implemented and their impacts on health care environments. This is particularly important as health systems develop future pandemic management plans and work on managing increases in communicable diseases, such as the global rise in measles infections [34].

Existing literature on hospital-based DCT during the COVID-19 pandemic has been primarily quantitative in nature, reporting on the risk of COVID-19 spread, retrospectively describing the transmission characteristics, or using mathematical modeling designs to predict outbreak scenarios. Nevertheless, limited research has examined the perspectives of key stakeholders, directly involved in decision-making and use of this technology, in relation to DCT implementation, adoption, or impacts in a real-world setting [24].

### This Study

This study addresses this gap and assesses the implementation of a web-based DCT technology at a specialized pediatric hospital in Ontario. Specifically, we (1) identify the factors influencing the adoption of DCT, (2) highlight variations in perspectives across 3 key stakeholder groups concerning DCT impacts, and (3) provide benchmarking evidence for future pandemic preparedness.

## Methods

### Setting

The Children’s Hospital of Eastern Ontario (CHEO) is one of the 2 specialized acute care pediatric hospitals in the province of Ontario. It is located in the capital of Canada, Ottawa, and serves a catchment area of ≥500,000 children and youth per year, aged between 0 to 17 years, from the Champlain region, western Quebec, regions of Nunavut, and Northern Ontario [35]. CHEO employs >3000 staff and health care professionals and provides a comprehensive and broad range of pediatric inpatient and outpatient specialist services [35].

### Contact Tracing at CHEO

Using the secure and Health Insurance Portability and Accountability Act–compliant REDCap (Research Electronic Data Capture; Vanderbilt University) platform [36], CHEO implemented a digital surveillance technology (hereafter referred to as the DCT tool), which is aligned with validated measures from the Ontario Ministry of Health and Long-Term Care.

Upon notification of a positive HCW or patient, the Infection Prevention and Control Department and the COVID-19 Safety Team (CST) initiated contact tracing and subsequent case investigations. The CST, comprising nurse practitioners, registered nurses, and licensed practical nurses, focused on all staff-related COVID-19 management. This included sharing the link to the DCT tool to potentially exposed staff and conducting all necessary follow-up. The DCT tool assesses the risk of COVID-19 exposure by capturing (1) the staff’s demographic and institutional information, (2) case contact details, (3) proximity with the COVID-19 case, (4) aerosol medical procedures with potential contact, (5) use of personal protective equipment, (6) symptoms, and (7) COVID-19 testing and its outcomes (Multimedia Appendix 1).

Upon staff completing a risk assessment through the DCT, the embedded algorithm within the DCT tool determines the level of exposure, categorizing staff as high- or low-risk. Subsequently, through an automated email-triggered response, it sends staff guidance and recommended prevention measures (eg, isolation, testing, and return-to-work procedures) based on the information that they entered in the system.

The DCT tool was used from June 10, 2020, to May 4, 2023, accounting for approximately 11,604 completed risk assessments. In 75% of completed risk assessments, 75.2% (8726/11,604) comprised of staff who encountered probable contact with a patient who was COVID-19 positive, 20.9% (2425/11,604) involved probable contact with positive staff, and <4% (453/11,604) were related to potential encounters with positive caregivers. The inpatient medicine unit, the emergency department, the hematology department, and the COVID-19 screening team had higher rates of completed entries.

### Study Design

The Reach, Effectiveness, Adoption, Implementation, and Maintenance (RE-AIM) framework was first published by Glasgow et al [37] to facilitate knowledge translation. The RE-AIM framework is a planning and evaluation tool that is used to support the design, implementation, and evaluation of interventions in health care contexts [37]. This study followed an exploratory qualitative design using semistructured interviews. Using qualitative methods with the RE-AIM framework enables in-depth insights into the dynamics of implementation, such as how adaptations were made, who was involved in the change, and why changes were made, which can be beneficial for guiding improvements and facilitating knowledge translation [38]. Trustworthiness in this study was achieved using several strategies, including reflexive analysis, member checking, providing illustrative quotes, and following the COREQ (Consolidated Criteria for Reporting Qualitative Studies) guidelines [39-41] (Multimedia Appendix 1).

### Participant Selection and Recruitment

Using purposeful sampling and snowball sampling approaches [42], participants from 3 stakeholder groups were recruited based on their involvement with the DCT tool: health care administrators and managers (“implementors”) who were involved in conceptualizing and implementing the DCT tool; “occupational specialists,” who made decisions based on the data obtained from the DCT tool; and HCWs, including regulated health care professionals or hospital support staff, who had used the DCT tool as “end users.”

Recruitment occurred through direct invitation via email to the implementors and occupational specialists known to the research team, with subsequent snowball sampling. Given that the study coincided with the peak of the pandemic, the team faced considerable challenges in the recruitment process (eg, busy staff, burnout, absenteeism, etc). Specific to the end users, we engaged a member of the CST to facilitate identifying and inviting potential participants based on their access to the database of completed contact tracing investigations. Potential participants were able to contact the research team directly if they were interested in participating or provide their email addresses to the CST member. In the latter case, a delegated research staff member (BO) then contacted potential participants via email. To facilitate recruitment, participants in the end-user stakeholder received a CAD $20 (US $14.45) gift card as a token of appreciation.

### Data Collection

Interviews were conducted between December 2022 and April 2023 via a videoconferencing platform (Microsoft Teams; 20/21, 95%) or through telephone calls (1/20, 5%). Interview guides were tailored to match the role of each stakeholder group, informed by the RE-AIM framework [37,38] (Multimedia Appendix 1). The interview guide explored the following topics: (1) the DCT tool’s reach (eg, willingness of HCWs to use the DCT tool), (2) its generated outcomes and perceived effectiveness (eg, positive and unintended), (3) the facilitators and barriers to organizational-level uptake, (4) the DCT tool’s implementation processes and challenges, and (5) the factors facilitating and hindering the sustainability of the DCT tool. The guide was used flexibly with probing questions to obtain clarification or gather richer data. Interviews lasted on average 33.6 minutes (range: 13 to 49 min) depending on the participant’s time and experience with the DCT tool. There was no preferred sample size; however, recruitment ceased after 21 interviews. At this point, all individuals who had expressed interest in participating had been interviewed, and the emerging codes and themes had become repetitive. All interviews were digitally audio-recorded and transcribed verbatim using Otter.ai. All participants received a summary of the findings for validation and member-checking purposes.

### Data Analysis

Interview transcripts were analyzed by applying principles of thematic analysis [40], to identify initial codes and patterns within the data, which then informed the organization of categories and themes. NVivo (QSR International) was used to code the data. Familiarization was undertaken by 2 researchers who independently coded a subset (6/21, 29%) of transcripts. Given the predominantly inductive nature of this research, a line-by-line coding method was used initially to generate preliminary ideas and to represent important concepts in the data set, which allowed the research to remain open and exploratory [39]. The researchers met to discuss the coding approach and disagreements, which were resolved through consensus. This led to the creation of an initial codebook, which was later refined as data analysis progressed. Specifically, codes that had similar meanings were combined, and related codes were then organized into several categories, which formed the initial themes. Next, a deductive approach was adopted, which involved using the RE-AIM framework to organize and produce themes that were meaningful to the research questions. The final codes and themes were then used to code and analyze all the transcripts. An iterative and systematic approach was used to develop and refine themes [40]. The final codes, categories, and themes are presented in Tables S1-S4 in Multimedia Appendix 1.

### Ethical Considerations

This study was approved by the research ethics board at CHEO and the University of Ottawa (approval number 22/114X). After eligibility was determined, participants were emailed the study’s cover letter and an electronic version of the consent form, which detailed the nature of the study and potential risks, stating that participation was voluntary, and refusal would not impact their employment. Participants provided an attestation of consent in the form of a signed statement via the electronic consent form. Before commencing the interview, participants were asked to confirm their name and email address, and to confirm that they signed the consent form themselves to authenticate their signature. Participants were provided an opportunity to ask the lead author (BO) questions before, during, or after the interview. The findings below include participant quotes that are anonymized and deidentified to protect participants’ confidentiality.

## Results

### Overview

Out of 27 persons who originally expressed interest in participating in this study, 1 (3%) was deemed ineligible (attributed to limited recall in using the DCT tool), and 5 (19%) were lost by attrition (unreachable for interview purposes). A total of 21 participants completed the interviews, resulting in a retention rate of 77% (21/27). Table 1 presents the characteristics of the participants.

**Figure 1:**
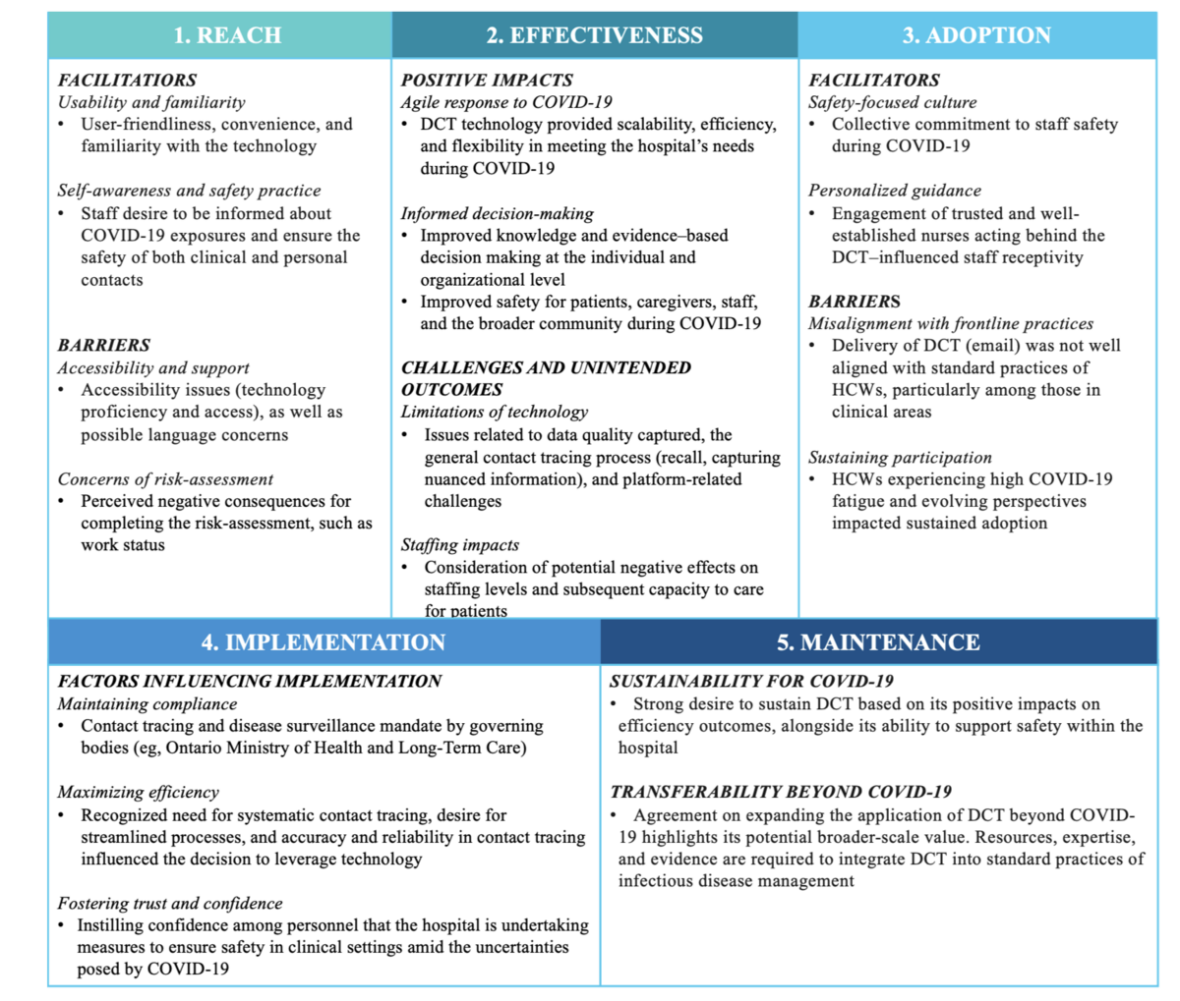
Summary of key findings: hospital stakeholders’ perspectives on digital contact tracing (DCT) during the COVID-19 pandemic at a children’s hospital. HCW: health care worker.

Figure 1 provides an overview of the findings aligned with the RE-AIM dimensions. In addition, the key themes identified in the findings, along with additional participant quotes, are provided in Multimedia Appendix 1.

**Table 1:** Overview of the groups and characteristics of participants or groups involved in the semistructured interviews (n=21) conducted at the Children's Hospital of Eastern Ontario.

Stakeholder groups	Description	Participants invited, n (%)	Interviews, n (%)	Participant characteristics
Implementors^a^	Administrative personnel involved in conceptualizing and implementing the DCT^b^ tool.	6 (7)	6 (29)	3 managers, 2 coordinators, and 1 vice president
Occupational specialists	Occupational health and safety specialists who make decisions based on the data obtained from the DCT tool.	14 (16)	8 (38)	5 registered nurses, 1 registered safety professional, 1 physician, and 1 registered practical nurse
End users	End users of the DCT tool	66 (77)	7 (33)	3 registered nurses, 2 physicians, 1 patient service clerk, and 1 family resource worker

^a^Some participants in the implementor group had dual roles as occupational specialists. As they were identified as being heavily involved in the implementation process, they were considered part of the implementor stakeholder group for the purposes of this study.

^b^DCT: digital contact tracing.

### Reach

#### Overview

“Reach” refers to the number, proportion, and representativeness of individuals willing to participate in an intervention [37]. As reported by participants, the DCT tool was directed to any CHEO personnel (eg, learners, volunteers, support staff, and medical staff) working within the same department, unit, or proximity to an identified positive COVID-19 case. For the purposes of the results, the collective group targeted by the DCT tool will hereafter be referred to as “staff.”

#### Facilitators

##### Usability and Familiarity

Implementors and occupational specialists agreed that the primary target of the DCT tool was generally frontline medical staff. When discussing the reach of the DCT tool, stakeholders referred to the higher convenience and user-friendliness of the technology relative to traditional contact tracing approaches, as factors influencing the program’s ability to engage the target population. Similarly, this perspective was observed among end users who characterized the DCT tool as “intuitive” and “straightforward,” signifying a preference for the use of the technology-enabled approach:

It worked out for me because I am not really good at answering my phone...The fact that it was emailed to me and that the questions were very brief, they were quick. That was pretty easy.

Participant 13, end user

Despite the varying nature of the hospital’s workforce (eg, education, age, comfort with technology, etc), stakeholders consistently conveyed that the DCT tool was perceived as user-friendly. Specifically, end users appreciated the DCT’s ability to adapt to mobile devices and the ability to provide supplementary details regarding their potential exposures via the embedded functionality (eg, comment boxes):

During the real pandemic when it first started, I was in ICU when we had idled COVID patients. And what I loved about it is, our senior nurses, which can’t stand anything technology-based, we’re like, “it’s so easy to navigate.”

Participant 18, end user

Stakeholders also acknowledged that staff’s familiarity and consistent use of the DCT tool influenced engagement. While implementors and occupational specialists expressed that similar digital screening used during the pandemic (eg, entrance screening) influenced familiarity, an end user credited their familiarity to the frequent use of the DCT tool because of the high prevalence of COVID-19 exposures in their clinical department:

The tool itself was easy to use. Because you click the link to REDCap, which we’re all very familiar with in the emerge.

Participant 01, end user

##### Self-Awareness and Safety Practices

The presence of fear and uncertainty among staff during the early stages of the COVID-19 pandemic was also reported as a facilitator increasing the reach of the DCT tool. However, no end user explicitly mentioned a connection between their fear and their use of the DCT tool. Instead, protecting the well-being of those in their working environment and concerns about transmitting the virus to family members were the driving factors:

So, I was the runner during the pandemic. And I wanted to make sure that I wasn’t bringing it home to my parents.

Participant 17, end user

Many implementors and occupational specialists also emphasized that the reach of the DCT tool was influenced by a sense of “professional responsibility.” An implementor stated, “...dedicated HCWs worry about their patients and would not want to inadvertently transmit something” (Participant 07, implementor).

An end user also echoed this sentiment, acknowledging their responsibility to contribute to organizational COVID-19 exposure tracking:

And I know that I work at CHEO, and it’s pretty easy, right? I’m in a setting where I get a tool delivered, and I can answer it, and I help the organization track.

Participant 01, end user

#### Barriers

##### Accessibility and Support

Participants reported minimal barriers hindering the DCT tool’s reach. However, they recognized the potential barriers related to staff’s access to smartphones or internet-enabled devices. This concern was raised not only by end users, but also emphasized by a few implementors and occupational specialists who expressed the following: “*...*a small minority of staff [did not] have cell phones” (participant 02, implementor). Consequently, this was perceived to impede some staff members’ engagement with the DCT tool, with a participant stating, “If you’re not inclined to have a smartphone, then obviously you can’t access it from there” (participant 14, end user).

Inconsistencies in stakeholder perspectives existed regarding the support available for staff with potentially lower levels of digital literacy. While end users indicated limited availability of live assistance when completing the DCT tool, implementors and occupational specialists reported that the CST was reachable through phone calls to support staff navigating issues:

I guess from a barrier standpoint, there isn’t a 1-800 number that you can call to have somebody do it with you online...So, there isn’t that in terms of an accessibility option there.

Participant 14, end user

We give them a phone call and then help them navigate. Sometimes people had to be helped navigate through these questionnaires. I think that can be a barrier... But we had people seven days a week.

Participant 15, occupational specialist

Participants from all stakeholder groups also acknowledged the presence of potential language barriers. While occupational specialists confirmed the availability of the DCT tool to be completed in English and French, an end user mentioned being unaware of this option. In addition, it was highlighted that nonclinical end users may have experienced health literacy barriers related to the complex contact tracing terminology within the DCT tool:

I guess, from a language point of view, if it’s in English exclusively, and people would like to fill it in French.

Participant 13, end user

We did receive feedback at some point that when we were trying to screen volunteers and do a risk assessment on volunteers, they didn’t understand the lingo.

Participant 11, occupational specialist

##### Concerns Regarding Risk Assessment

Participants from all stakeholder groups characterized the DCT tool as an “honor system,” as staff were accountable for accurately completing their risk assessment while also being aware of the implications of being identified as a high-risk contact. For instance, this was perceived to involve potential financial constraints, concerns about attendance programs, or other negative repercussions. Interestingly, while none of the end users identified these factors as barriers to their engagement with the DCT tool, several participants among stakeholder groups anticipated these barriers existed among staff:

I guess, if I put on somebody else’s shoes and think about what they might feel, maybe they worry about breach of confidentiality. Maybe they worry about identifying themselves as not having utilized all the equipment that they were supposed to be using in the room at the time or with a particular colleague. And that might put them out of work, right?

Participant 01, end user

### Effectiveness

#### Positive Impacts

##### Agile Response to COVID-19

“Effectiveness” involves assessments of outcomes related to the impacts of an intervention [37]. There was overwhelming agreement among stakeholders that the pandemic necessitated rapid changes within the hospital, including various modifications to surveillance policies and procedures. Implementors and occupational specialists extensively discussed the “ever-changing” guidance on contact tracing. Participants also perceived that the DCT tool provided an adaptable response to COVID-19 by quickly integrating these guidance changes:

We were basically just tweaking our tool. It was always, “Let’s review this information that’s provided from the provincial guidance” and cross-reference it to our decision tree, and then confirm whether or not we had to make changes, and what changes needed to occur, if we needed new alerts, or if it was just a matter of changing, or adding more information to our current alerts.

Participant 02, implementor

A central concept highlighted by various stakeholders was the pivotal role of the DCT tool in optimizing the use of HHR during a period of resource constraints. The DCT tool enabled the CST team to leverage their clinical skills for a “more meaningful and impactful human interaction perspective” (P13, end user). Specifically, participants noted that by avoiding repetitive phone calls, it enabled the CST to use their skills and expertise when completing case investigations and monitoring exposed staff. In addition, other benefits included the elimination of “monotonous” tasks related to traditional contact tracing approaches, reducing the need for numerous “touch points,” and lowering the number of HHR required to support infectious disease monitoring during the pandemic:

Our initial exposure investigations involved dozens to hundreds of healthcare workers. You’re trying to do a phone call to ask the same questions, I think that would have been so inefficient and not a good use of the skills and expertise that the occupational safety nurses have.

Participant 09, occupational specialist

And then of course, the organization benefited financially, in the sense that we didn’t have to have 20 or more COVID nurses. Or do this as a manual process, right?

Participant 02, implementor

Implementors and occupational specialists also considered a key advantage of the DCT tool was its “timeliness” and capacity to “...reach [a] large number of people in a short span of time” (participant 04, implementor). In turn, this facilitated the CST to gain a “broad capture” of the possible transmission dynamics:

Initially, we were really thorough, like, “Okay, this patient was positive in emerg and 47 people saw them on these days.” Plus, we would go into the nursing students that were on the floor, or if there was an RT [respiratory therapist] student that was with another RT. We were super thorough, and then we were able to capture everyone...And definitely, we were able to get a bigger picture than any other hospital with this.

Participant 21, occupational specialist

Furthermore, implementors and occupational specialists valued the DCT tool for offering continuous check-ins and updated information to staff, eliminating concerns about finding the latest guidance for returning to work protocols. This “just-in-time” guidance was also valued by end users, as compared with traditional contact tracing approaches, where delays frequently occurred:

It was quick. It would tell me what my next steps were. Whereas in the paper form, it was like, “Someone will get back to you within 24 hours.” So, you might be sitting there spreading everything, but you didn’t know [what] your next steps were supposed to be. That’s why I think it was so beneficial.

Participant 18, end user

##### Informed Decision-Making

By offering reproducible contact tracing measures throughout the hospital, several participants recognized that the DCT tool enabled evidenced-based decision-making, at both the individual and organizational levels. At the individual level, participant stakeholders shared the perspective that the DCT tool supported staff education. End users also perceived this to influence personal decision-making, such as recognizing instances of elevated COVID-19 risk, understanding the need for extra precautions, or remaining under self-isolation protocols:

It just helps you sort of make the decision, like, “Okay, I was a high risk. And I meet these criteria, and I will be sent the next steps for me to do.” Or “I wasn’t there for very long...” It helps you make your own decision and your own risk assessment.

Participant 20, end user

Stakeholders also expressed that the DCT tool had a comprehensive impact on safety during the pandemic, including enhancing staff well-being and protecting secondary exposure to their personal families. In addition, they reported that the DCT tool enabled protection for patients and their caregivers. However, it was also acknowledged that it extended beyond its immediate application in the hospital and was perceived to contribute to a reduced rate of infection in the community:

The primary benefit would have been for [staff] themselves and their families. But the secondary benefit would have been for protecting their patients, that they were coming to work to take care of.

Participant 07, implementor

One, the patient. Two, the families. Three, the staff. And four, our community. Because we are saying that we are a safe environment, we have these measures in place. But not only are we saying it, but we’re using tools in order to prove that we have those measures in place.

Participant 16, end user

At the organizational level, implementors and occupational specialists noted the crucial role of these data in tracking transmission rates and assessing hospital-acquired infections. This positively influenced the hospital’s safety culture, as access to real-time data enabled hospital leaders to make well-informed decisions to improve infection control measures:

I think the tool was so successful with the contact tracing... Organizationally, it gave us a great idea of where 30% of the cases that turned positive, [it was] because those people had a breach in their PPE, or they didn’t wear PPE. Or, you know, this and that.

Participant 06, implementor

Implementors and occupational specialists noted the DCT tool’s contribution to the hospital’s ability to achieve top regional safety performance during the pandemic. Specifically, these participants emphasized that by promptly detecting and managing potential exposures, the hospital had a limited number of nosocomial outbreaks among staff and patients:

If you look at the number of outbreaks and everything like that. That’s the number one “why” we kept it at CHEO. That tool was able to help keep CHEO safe from COVID and have the best COVID safety record in the region. So, I think that’s huge. What we did worked.

Participant 19, occupational specialist

Interestingly, some participants acknowledged that the low number of hospital-acquired infections could not be solely attributed to the DCT tool. Implementors and occupational specialists emphasized the influence of other COVID-19 prevention measures that collectively provided “*...*layers of protection” to ensure staff and patient safety:

I think that it works, what we did was working. And plus, it’s not only the contact tracing tool. But there were also other mitigations that we had in place. Our screening was very intense. Our masking, everything was.

Participant 19, occupational specialist

#### Challenges and Unintended Outcomes

##### Limitations of Technology

Several challenges and unintended outcomes were noted by the respondents. Implementors and occupational specialists perceived that the absence of interactive engagement between the staff and CST (eg, phone interviews) limited the DCT tool’s ability to capture “nuanced” information. In turn, this impacted the CST’s ability to actively probe staff on follow-up questions or seek real-time clarifications:

I think getting a precise understanding of the types of exposure [staff] had is really challenging virtually. When you’re having a conversation with someone, you can ask follow-up questions, you can ask clarifying questions. When you’re just reading what has been submitted, you’re depending on the person writing to have precise language and a good understanding of what’s being asked. I think that’s probably the biggest challenge is that for us interpreting it, we don’t always get the best picture. Or as clear a picture as we would have been able to get verbally.

Participant 05, occupational specialist

Although not directly tied to the DCT tool, occupational specialists also pointed out that the contact tracing processes, particularly the EMR review, were limited in capturing all personnel who may have been at risk of COVID-19 exposure. This challenge was particularly related to distinguishing the staff who were present during the period of communicability with the positive case, as opposed to those who simply accessed the EMR to review a patient chart. This was perceived to impact the precision of the DCT approach:

If you are seeing [a patient] or if you have something to say, there’s charting that will be done. Versus, “Yeah, I was working in pharmacy that day. I sent off an email, but I was never in the room.” There are certain people that are just in the chart to look at the information or to write a note, they have never seen the patient and never were in the room.

Participant08, occupational specialist

The respondents also recognized that during periods of elevated COVID-19 transmission, staff frequently experienced delays in receiving or completing the DCT. As a result, participants across the stakeholder groups recognized the recall limitations with contact tracing. An end user expressed additional insights on this challenge, noting the challenges of not having sufficient “personal health information*”* (eg, contact information), they were unable to accurately record all potential exposures in the tool:

Sometimes [the] clinical staff were being notified three or four days after their shift. They were like, “Which one of the five kids? Or, the additional two that I covered for my colleague? I can’t remember which one you’re talking about.” Or “Yeah, they were 12. But were they masked every time? I don’t know, because I wasn’t asking them to mask every time I went to see them.” Or “Did I go close to their face for a prolonged period of time? I don’t know.”

Participant 09, occupational specialist

Although an infrequent occurrence, implementors and occupational specialists described that the functionality of the DCT tool (eg, free-text fields) led to scenarios in which incomplete or inaccurate data were captured by the DCT tool. In turn, this impacted staff’s ability to receive subsequent guidance on their exposures:

It asks [for] your email, and it asks for your manager or supervisor’s email. The only way to have those populated, is that you manually have to type in that field. So, if you type in that field wrong, it’s automatically not going to send. You don’t get another chance to go back in and edit it... So, you’re never going to get that bounce-back email to you about what to do next.

Participant 06, implementor

Implementors and occupational specialists also indicated that using the same platform for both entrance screening and contact tracing resulted in the platform being overutilized. While also occurring infrequently, this occasionally led to the creation of new links to the DCT tool.

##### Staffing Impacts

A subset of stakeholders highlighted the DCT tool’s potential unintentional impacts on staffing. A few occupational specialists suggested that while the contact tracing efforts taken by the hospital aligned with safety precautions, the wide distribution of the DCT may have potentially led to challenges in staff management:

I think that one outcome is that we possibly overdid the amount of contact tracing that was necessary...So, sometimes when we do a contact trace there can be over 100 staff that were in contact with the patient, for example. We’re sending all of them the survey to complete. In some ways, I think that the tool may have been almost too broad, and not specific enough to understand who is actually at risk.

Participant 05, occupational specialist

In addition, guidance from public health authorities restricted staff from entering hospitals after exposure to COVID-19. As a result, staff were occasionally off duty for 10 to 14 days, with this time being extended for those who contracted COVID-19 during their isolation period. This situation was also perceived to exert additional pressure on staffing, posing a challenge to the hospital’s capacity to deliver patient care:

They’re off for five days or four days, and then they got COVID. Then, they were out for another 10 days. So, that put the unit out two weeks from medical staff or other staff.

Participant 09, occupational specialist

Additional insights into system-level challenges impacting staffing were reported, including the consequences of an overburdened health care system exacerbated by the COVID-19 pandemic:

And then, it would impact the [patients] negatively. Because then it would be, if we had to isolate staff, they couldn’t come into work. Then, there was no staff to take care of the patients. We’re short-staffed already, in an already stretched healthcare system.

Participant 19, occupational specialist

#### Perceived Effectiveness Indicators

When asked about the assessment of DCT effectiveness in a hospital setting, participants provided a set of high-impact indicators that may be used as benchmarks for measuring perceived effectiveness (Textbox 1).

**Perceived indicators of digital contact tracing (DCT) effectiveness by stakeholders at the Children’s Hospital of Eastern Ontario.**

**Responsiveness and engagement**

- Efficiency in reaching target audience: evaluating if a DCT has the capacity to consistently reach the target audience.
- Timeliness of delivery: examining the speed at which DCT is deployed to its target audience.
- DCT versus traditional contact tracing approaches: analyzing the timeline of case investigations when using DCT technologies compared with traditional contact tracing.
- Staff satisfaction with DCT: measuring staff satisfaction on the use of DCT.
- Staff confidence in DCT: measuring staff members’ confidence in the hospital’s safety mitigations that were facilitated by DCT.

**Infection indicators**

- Symptom management: investigating if DCT enables staff to monitor symptoms more effectively, subsequently leading to a reduction in transmission.
- Outbreak prevention: determining the extent to which DCT contributes to preventing outbreaks.
- Risk mitigation: evaluating if DCT helps to enhance or decrease safety mitigations.
- Accuracy of risk assessment: evaluating the reliability and accuracy of DCT for identifying high-risk individuals.
- Patient outcomes: evaluating if the DCT had any associated impacts on patients.

**Technical operations**

- Flexibility and adaptability: evaluating if the DCT can be adapted to make changes in response to evolving needs.
- User-friendliness: assessing if the technology is user-friendly for its intended audience.
- Transparency: evaluating if the outcomes of the DCT are clear and transparent for the users.
- Data storage and reporting: assessing the technology’s capabilities to store and share data.

### Adoption

#### Facilitators

##### Safety-Focused Culture

Within the RE-AIM framework, “adoption” focuses on the willingness of settings and intervention agents to initiate a program, considering factors, such as number, proportion, and representativeness [37]. In our study, we aimed to gain a nuanced understanding of the factors influencing successful DCT adoption at the setting-level, specifically by exploring the facilitators and barriers.

Across implementors and occupational specialists, the prevailing thought was that the implementation of the DCT tool was “well-received” and “widely adopted” by most of the staff. Participants provided insights into safety-focused strategies that influenced the integration of the DCT tool into routine practices, including regular internal communications on COVID-19 policies and adherence to organizational work standards during the pandemic:

And it was also communicated to staff, “This is how CHEO is going to be doing contact tracing. We’re not going to phone; we just can’t phone everyone anymore. So, this is what you can expect.”

Participant 03, implementor

Implementors and occupational specialists perceived the provision of COVID-19 pay incentives and the assurance that COVID-19 absences would not negatively impact attendance records as factors contributing to staffs’ uptake:

During the pandemic, with the knowledge and understanding that if you were sick, there was pandemic pay. So, if you were off related to COVID, then you were paid. So, there was less hesitancy to report. And then since you were (1) being paid and then (2) the rule was that COVID-related absences wouldn’t count toward the attendance program. So, if you were sick for several weeks because you had contracted COVID, you wouldn’t be penalized for those absences.

Participant 02, implementor

##### Personalized Guidance

Many participants across stakeholder groups recognized the crucial role played by the CST in promoting the adoption of the DCT tool among staff. Specifically, implementors noted that the trusted relationships that staff had with nurses overseeing the DCT tool played an impactful role:

The staff that they had doing the COVID contact tracing, were really senior, well respected, sensible nurses, that people really trusted. I think once they appreciated that the people that were going to follow up with them, that were behind the scenes, there was really a real-life human being, and that was nurses that they knew and that they trusted.

Participant 07, implementor

Similarly, occupational specialists emphasized that their “direct touch” with the DCT tool alongside their *“critical care”* background facilitated staff to be more receptive to the technology. They also highlighted that the CST demonstrated a collective commitment to maintaining a high standard of contact tracing during the pandemic:

We really tried to maintain a high standard. And we were all, as a team, willing to maintain that high standard. There was nothing ever laissez-faire, or we could just do it. Or we’ll just pop this out. It was always a high standard.

Participant 11, occupational specialist

#### Barriers

##### Misalignment With Frontline Practices

As perceived by implementors and occupational specialists, the primary barrier impeding the adoption of the DCT tool was the culture of email use among frontline HCWs, ie, the challenge of creating awareness among staff regarding consistently checking their emails. This was further challenged by staff choosing not to receive or engage with communications from the hospital during scheduled days off, working on rotating shift schedules, and lacking access to their hospital email on personal devices:

Frontline staff are the people in the building, who use email the least frequently. Or they do not. Some of them are off, some of them do not get it on their phone. Especially maybe the...well, I would not want to generalize. There’s a portion of the population that just does not get emails on their phone. And also think, “I’m not going to check work emails when I’m not working. I’m off for three days, I don’t want to hear from CHEO, I don’t want to think about CHEO, nothing.”

Participant 03, implementor

Several implementors and occupational specialists discussed the hospital’s multidisciplinary nature, particularly regarding how different health care professionals engage with email in their daily practices. They perceived a misalignment between the delivery of the DCT tool and the standard practices of HCWs, as their job responsibilities typically do not involve frequent email checking and some staff may have unintentionally disregarded the DCT tool because of the sheer volume of organizational emails:

I have some colleagues, or just people that I know who, when I look at their little notification button on their email there’s, like 246. So, I can imagine that in there, you could potentially have missed something.

Participant 01, end user

##### Sustaining Participation

Sustained adoption of the DCT tool was challenged at 2 levels as reported by implementers and occupational specialists. First, staff’s perceived threat of exposure declined as the pandemic continued and as preventive practices within the community declined:

At the beginning. I feel like...again, this is what I've heard from different people. [The staff] really wanted to be made aware, because then that could potentially affect other people that they would go see. Or some people do work in different locations, different hospitals, so they would up their PPE when they were working at their other job. And now they're just, “Since there's no restrictions in the community, then why am I still getting surveys?”

Participant 08, occupational specialist

Second, frontline HCWs’ heightened workload, increased burnout, and heightened levels of contact tracing fatigue contributed to rates of noncompliance over the duration of the pandemic. It was noted that as the COVID-19 situation improved and with the widespread distribution of the DCT tool, completing the risk assessment became *redundant* or *white noise* for staff:

So, I know, emerg was constantly getting the tool because they’re constantly exposed. Because obviously,... it’s less of a controlled area. The volume of people with kids that they’re seeing is so much higher than other units, right? So, they were constantly getting blasted with the digital tool. So, they were over it. They were not filling it out by the end or deleting it. They were just filling it out, just to fill it out.

Participant 19, occupational specialist

While none of the end users expressed such perspectives explicitly, some underscored the increased demands of completing electronic surveys during the COVID-19 pandemic. Although this sentiment primarily arose from the daily screening requirements, it does highlight the parallel strain imposed on staff being required to complete repetitive survey tasks. In addition, several implementors and occupational specialists believed that challenges related to staff’s fatigue with the DCT tool did not indicate a lack of success. Instead, they acknowledged that even with traditional contact tracing approaches, staff would likely face similar fatigue-related issues due to the frequency of required completions during the pandemic:

I think they would have been just as fatigued with the phone call; it wasn’t the survey itself that was the problem. It was the number of times that they had to fill it out, that was the problem.

Participant 03, implementor

#### Implementation

While the fidelity of an intervention is commonly quantitatively measured [37], our study aimed to offer nuanced insights into the system-level factors and contextual pandemic-related determinants shaping the implementation of the DCT tool.

First, implementors described that government mandates necessitated clinical environments to adapt to new regulations for managing infectious diseases. Most of them viewed the implementation of DCT as a tool to meet organizational responsibilities and ensure adherence to COVID-19 protocols, although one occupational specialist considered it a “follow-suit” action:

I think if public health didn’t do it, I don’t know if we would have started to do it. We sort of follow what they do, they come out with their guidance, and we just follow what the guidance says.

Participant 10, occupational specialist

Second, several implementors and occupational specialists expressed that staff anxiety, concern, and fear of COVID-19 exposure posed challenges, such as transmitting the virus to those in their personal and working environments. This underscores the importance of building trust and fostering a supportive workplace culture. As such, establishing this *transparency* among the staff played a pivotal role in the implementation of the DCT tool:

The number one internal thing. Well, internal and external, people were scared, right? People were really, really scared at the beginning of COVID. We didn’t really know what this thing was, we didn’t know how it was transmitted, and we didn’t know how sick people were going to get. People were scared to come to work. It was really, really important to build trust among our staff, that we were doing all we could to protect them. So, that they could feel safe coming to work. That was one of the primary sorts of internal reasons for doing this. We knew sick people would be coming into the hospital.

Participant 12, implementor

Finally, traditional contact tracing methods faced limitations, prompting the exploration of alternative approaches. The limited availability of resources, specifically HHR, played a crucial role in shaping the decision to leverage technology for contact tracing. Furthermore, the time-sensitive nature of responding, the scale of contact tracing, and the need for *standardized* processes drove the motivation for the implementation of the DCT tool:

I know that there was a lot of pressure on the system. That would have resulted in huge delays, and in contact tracing that would have rendered it quite meaningless if there hadn’t been a way to sort of speed it up. ... I would [say] that from a leveraging technology standpoint, the ability to do it with less people, and quickly, and to get meaningful data that was standardized, to get that back in real-time. I think would have weighed heavily on the decision to go ahead and implement it.

Participant 07, implementor

### Maintenance

#### Adaptations and Modifications

“Maintenance” reflects how well a newly implemented intervention is integrated, institutionalized, or sustained within a setting [37]. In this study, we explored how the DCT tool was successfully institutionalized at the hospital level, alongside stakeholders’ perspectives on the long-term sustainability of this technology.

Various factors supported the sustained use of the DCT tool during most of the pandemic. Implementors and occupational specialists emphasized the relevance of a “continuous improvement” approach, in which feedback was actively solicited from various stakeholders. For instance, the CST implemented weekly meetings for this purpose, and end users were encouraged to provide suggestions in an embedded commentary box at the end of each survey:

We would have weekly touch base meetings, so that we could talk about, not just this survey, it was all REDCap things. What can be changed to either make the workflow more efficient? At some point, we developed standard responses. Like drop-downs and standard responses that [staff] could select, as opposed to all free text. But [the CST] were involved all along, in order to really make sure that we were improving the tool as we went.

Participant 03, implementor

Apart from enhancing the efficiency of the DCT tool, several implementors and occupational specialists emphasized the need for various adjustments in the delivery of the technology to adapt to the demands of transmission rates. Consequently, this resulted in a shift in accountability among staff to report potential exposures or breaches in personal protective equipment. In this scenario, the DCT tool was then distributed to these individuals. An occupational specialist perceived that this streamlined approach was less laborious and time-intensive, with no apparent impact on transmission rates within the hospital:

When we got slammed with Omicron, it was, we couldn’t do that anymore. Because we’re getting 30, 40, 45 new cases a day. So, we couldn’t just keep contact tracing that way. So, we ended up relying on the positive case itself, to let us know who they had contact with. And we only send it to those people. And then that was way better. And in the end, we found out it’s less work, it’s less people, and that it didn’t increase our high-risk contacts or increase transmission.

Participant 19, occupational specialist

#### Navigating Future Sustainability

Two key themes emerged on the long-term viability of the DCT approach: (1) sustainability of DCT for COVID-19 and (2) transferability beyond COVID-19. An in-depth summary of these themes is presented in Table 2.

**Table 2:** Stakeholders’ perspectives on the sustainability of digital contact tracing (DCT) at the Children’s Hospital of Eastern Ontario.

Key findings			Participant quote
**Sustainability**			
	Many stakeholders expressed the desire to sustain the DCT based on its positive impacts on efficiency outcomes, alongside its ability to support safety mechanisms within the hospital. A few stakeholders expressed that the practice of contact tracing was no longer valuable for staff based on HCWs’^a^ perceived diminished threat of being exposed to COVID-19. As such, these participants advocated for the discontinuation of the DCT tool.	“Well, we’ve had a COVID pandemic... and then we had the RSVb on the rise. I know even though it wasn’t for RSV, I’m just saying that having it allows us...it shows that we’re still keeping track. It’d be nice to keep it forever, for at least another while.” [Participant 17, end user] “It’s tricky, I don’t know what they’re going to do going forward because we can’t keep contact tracing for COVID, because no one cares anymore. Because it’s everywhere, it’s in the community. People aren’t dying like they used to be...I just don’t think it’s a big enough thing anymore that we need it for this.” [Participant 10, occupational specialist]	
**Transferability**			
	Stakeholders agreed on expanding the application of DCT beyond COVID-19, highlighting its potential broader scale value for managing various infectious diseases.	“But it would be easy to take the current tool and adapt it for all viruses or infections. It could be used for Ebola, it could be used for TB^c^, could be used for MERS^d^, any illness. And hopefully not the bird flu. But for any epidemic or infectious disease that may have public health concerns, the tool can easily be adapted to fit whatever is going on. So, even though it may go away for COVID, I imagine it would be used in other scenarios.” [Participant 04, implementor]	
	Stakeholders suggested that with the necessary resources, expertise, and evidence, DCT could be integrated into practices of infectious disease management. This included buy-in from staff and leadership, financial support, evaluations and assessments of its impact, and the availability of scientific evidence to inform evidence-based decision-making.	“It would need to be a project, it would need to have an infusion of support, enthusiasm, and probably money to be able to set up REDCap^e^ contact tracing for all of the things that they have to contact trace, but I could see that. Again, I think it’d be very leadership-dependent.” [Participant 07, implementor] “I guess it’s just the money thing. If there’s enough money to maintain the program. If there’s enough impact from current studies, current research, or current public health initiatives. Then, I don’t think there’s any reason why it would be difficult to maintain. I think it’s sustainable for sure.” [Participant 13, end user]	

^a^HCW: health care worker.

^b^RSV: respiratory syncytial virus.

^c^TB: tuberculosis.

^d^MERS: Middle East respiratory syndrome.

^e^REDCap: Research Electronic Data Capture.

## Discussion

### Principal Findings

While previous research has addressed the use of DCT technologies in other pediatric contexts during the COVID-19 pandemic [20,43], this study is the first to explore the perspectives of various stakeholders involved in pediatric care. Our findings reveal that there was notable alignment among stakeholders’ perspectives across the RE-AIM dimensions. Most respondents reported acceptance and adoption of the DCT technology among hospital staff during the COVID-19 pandemic, including medical and support staff. Health care leaders, occupational safety specialists, and frontline HCW’s indicated that the pandemic required rapid changes within the hospital, and the DCT tool was perceived as scalable and efficient for meeting the increased demands imposed on the health care system.

Our findings highlight that DCT, when used in the hospital context, also carries broader implications for safety within and beyond the hospital through improved COVID-19 awareness. The challenges identified in this study included the limitation of the DCT tool in capturing all nuanced information during probable positive interactions, technological limitations, including access and literacy, potential biases in responses, and the negative implications of contact tracing on staffing management. Despite these limitations, stakeholders agreed that the DCT tool could be scaled up and seamlessly integrated into the hospital’s standard infection control practices. Last, we extend the literature on the pragmatic use of RE-AIM by demonstrating a qualitative application of the framework in a digital health care context.

Before the COVID-19 pandemic, the health care sector lagged in technology adoption [44]. Among several other digital solutions, the pandemic has catalyzed the uptake of DCT in health care to augment contact tracing processes [6-8]. Most respondents shared comparable perspectives when describing the engagement with the DCT tool and how it was facilitated by HCWs’ self-accountability, desire to follow safety procedures, and working within a pediatric hospital. However, variations in stakeholder perspectives were evident surrounding the influence of COVID-19–induced fear on engagement with the DCT tool. While no HCWs explicitly cited fear to influence their engagement, several did express the desire to safeguard their personal health and the well-being of others, including their families, colleagues, and patients. A recent scoping review by Nashwan et al [45] reported that during COVID-19, HCWs were primarily concerned with contracting the virus, infecting family members, and caring for patients, all of which align with the results of this study.

Our findings suggest that the adoption of the DCT tool was generally high at the hospital level, contrasting what has been reported within the community setting during the COVID-19 pandemic [46]. We speculate this difference may be explained by the contextual factors of the hospital environment, including the hospital culture, HCWs’ professional accountability, desire for COVID-19 exposure awareness, and working among a pediatric patient population. While all stakeholder groups recognized the convenience of using technology for contact tracing, participants in this study also suggested a gap in communication regarding the availability of support channels for the DCT tool. Future initiatives using DCT should consider striking a support team to address the needs of stakeholders with varying digital literacy. It is also important to establish clear and accessible communication channels (eg, written materials, face-to-face communication, or self-service resources, such as videos or commonly asked questions), in addition to direct access to support via phone or email. Future research may consider using frameworks for digital health equity [47] during DCT implementation planning to best promote equal access and engagement among all intended users.

Overall, the use of DCT in a hospital context presented several positive outcomes, including promoting access to real-time information, supporting evidence-based decision-making, and enhancing resource efficiency. While our findings also echo that DCT can enhance infection control measures in clinical settings [10,18,23,48-50], this study goes further to suggest a secondary benefit of automated guidance associated with COVID-19 education and awareness that can positively influence the practices of HCWs. Subsequently, the DCT tool was perceived to contribute to a reduced rate of hospital-acquired COVID-19 transmission, in addition to positive safety implications for the broader community. These findings present a compelling case for integrating DCT approaches into pandemic preparedness policies to further enhance hospital resilience, improve infection control practices, and mitigate the strain on critical HHR during times of crisis.

While acknowledging the positive outcomes of the web-based DCT approach, our study also echoes challenges reported in the literature for both digital and traditional contact tracing approaches [9,18,20,21,23,51]. However, our findings further provide a more comprehensive understanding of the challenges involved with the use of DCT within the hospital context, stemming from engagement fatigue, recall biases, and accurately capturing the intricate transmission dynamics. Consequently, inaccuracies in the data collected by the DCT tool may have led to inaccurate risk assessments and adherence to infection control practices, thus presenting a limitation to this approach. In addition, a major finding is the misalignment of the DCT technology with the established practices and email habits of frontline HCWs, an area that should be addressed in future DCT technology implementation. This finding aligns with existing research on HCWs’ adoption of technology, indicating that perceived disruptions in workflows often present barriers to technology integration and adoption [52]. The decline in staff willingness to use the DCT tool in the later stages of the pandemic was also perceived as a barrier to long-term sustainability of this technology. This may have been attributed to the increased physical and mental demands placed on HCWs during the COVID-19 pandemic [53], the increase in community-acquired COVID-19 infections, and the discontinuation of public health measures [54]. Thus, we recommend that future hospital-based DCT approaches be codeveloped and tailored to meet the workflow needs of the intended users, thereby integrating into existing practices and sustaining rates of adoption.

Last, researchers have emphasized the importance of establishing a collectively agreed-upon set of indicators to facilitate comparisons and improvements related to contact tracing efforts [55]. The findings from this study hold unique insights, as we present stakeholders’ perceived indicators of DCT effectiveness, including (1) staff’s engagement and responsiveness with technology, (2) an extensive scope of infection indicators, and (3) technical operations. While some of these indicators are more applicable to the web-based DCT approach, they offer valuable guidance for both practice and policy considerations in the field of technological advances for HCW screening and surveillance. Researchers can build on these findings to inform the development of a practical framework for assessing the quality of DCT approaches.

### Limitations

This study is based on the experiences at a single hospital with a web-based DCT approach and therefore should be transferred with caution to other contexts. The rapidly evolving pressure on HCWs presented challenges with recruitment during the data collection process, which may have also affected the ability to recruit a broader pool of participants. Although we captured the perspectives of participants involved with the DCT technology in different capacities, the study sample may not be representative of all hospital stakeholders. Building on this exploratory qualitative research, future studies can develop a multi-site, broader project, with diverse medical professionals, that assess the factors that emerged in this research, which can influence DCT adoption and success. Recall biases may also have influenced this research as data collection was conducted in the middle of the pandemic after some time since the implementation of the DCT tool. In addition, we acknowledge that we reported on clinical areas with high use rates of the DCT tool, but we have not provided corresponding information on the areas with lower use. Last, some of our findings may not be exclusive to the application of DCT, but instead to the broader practice of contact tracing in the hospital context. Future studies may wish to consider understanding and distinguishing the outcomes specifically related to DCT approaches from traditional approaches.

### Conclusions

Grounded in the RE-AIM framework, this study is the first to draw on the perspectives of various hospital stakeholders to understand the adoption, implementation, and impact of a hospital-based DCT approach during the COVID-19 pandemic. Health care leaders, occupational health and safety specialists, and frontline HCWs converged on the fact that DCT is an acceptable and efficient approach for identifying and monitoring COVID-19 cases. In addition, most stakeholders perceived that DCT forms the foundation of standard infectious disease practices beyond the pandemic, though technological challenges and misalignments in the tool’s delivery were noted. Considering the advantages reported, the use of web-based DCT approaches is recommended in complex health care environments. As COVID-19 transitions into an endemic phase, the lessons learned over the last 2 years will be crucial in preparing and planning for future epidemics and pandemics [56]. This is particularly relevant as we are witnessing a global rise of returning communicable diseases, such as measles [34,57]. Collectively, our findings contribute to the literature on digital innovations for disease surveillance and present benchmarking results that can inform health care practice, policy, and future studies.

## Appendix

Multimedia Appendix 1 Implementation of the digital contact tracing tool during the COVID-19 pandemic and experiences and perspectives of hospital leaders.

## Abbreviations

CHEO: Children’s Hospital of Eastern Ontario
COREQ: Consolidated Criteria for Reporting Qualitative Studies
CST: COVID-19 Safety Team
DCT: digital contact tracing
EMR: electronic medical record
HCW: health care worker
HHR: human health resource
RE-AIM: Reach, Effectiveness, Adoption, Implementation, and Maintenance
REDCap: Research Electronic Data Capture
