# Supplementary material for: Digital Contact Tracing Implementation Among Leaders and Health Care Workers in a Pediatric Hospital During the COVID-19 Pandemic: Qualitative Interview Study
Source: JMIR Public Health Surveill. 2024 Nov 5;10:e64270. doi: 10.2196/64270 (PMC11576617; doi:10.2196/64270)
Supplement: Multimedia Appendix 1 [file publichealth_v10i1e64270_app1.docx]

Supplementary Material


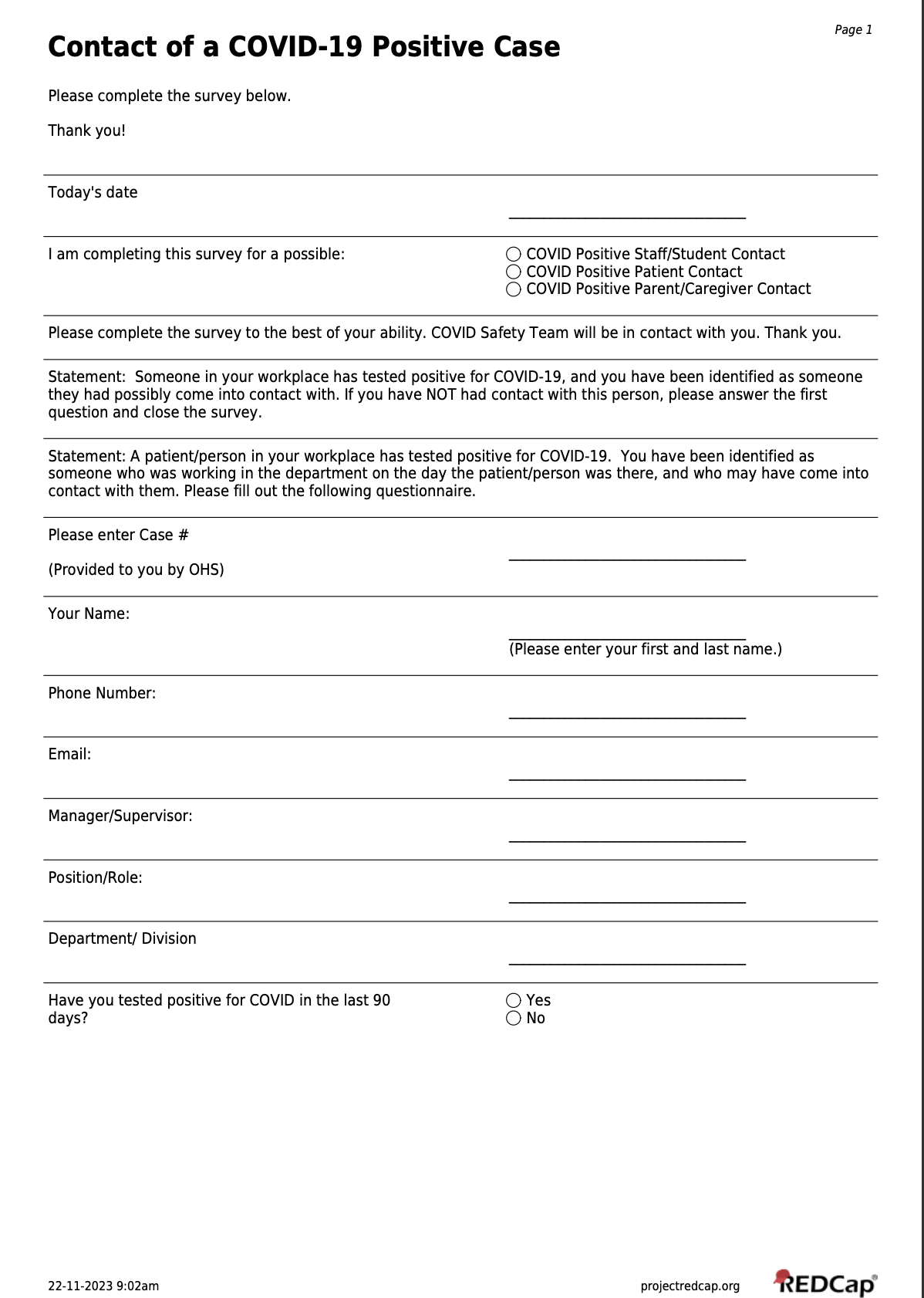
Digital Contact Tracing Tool


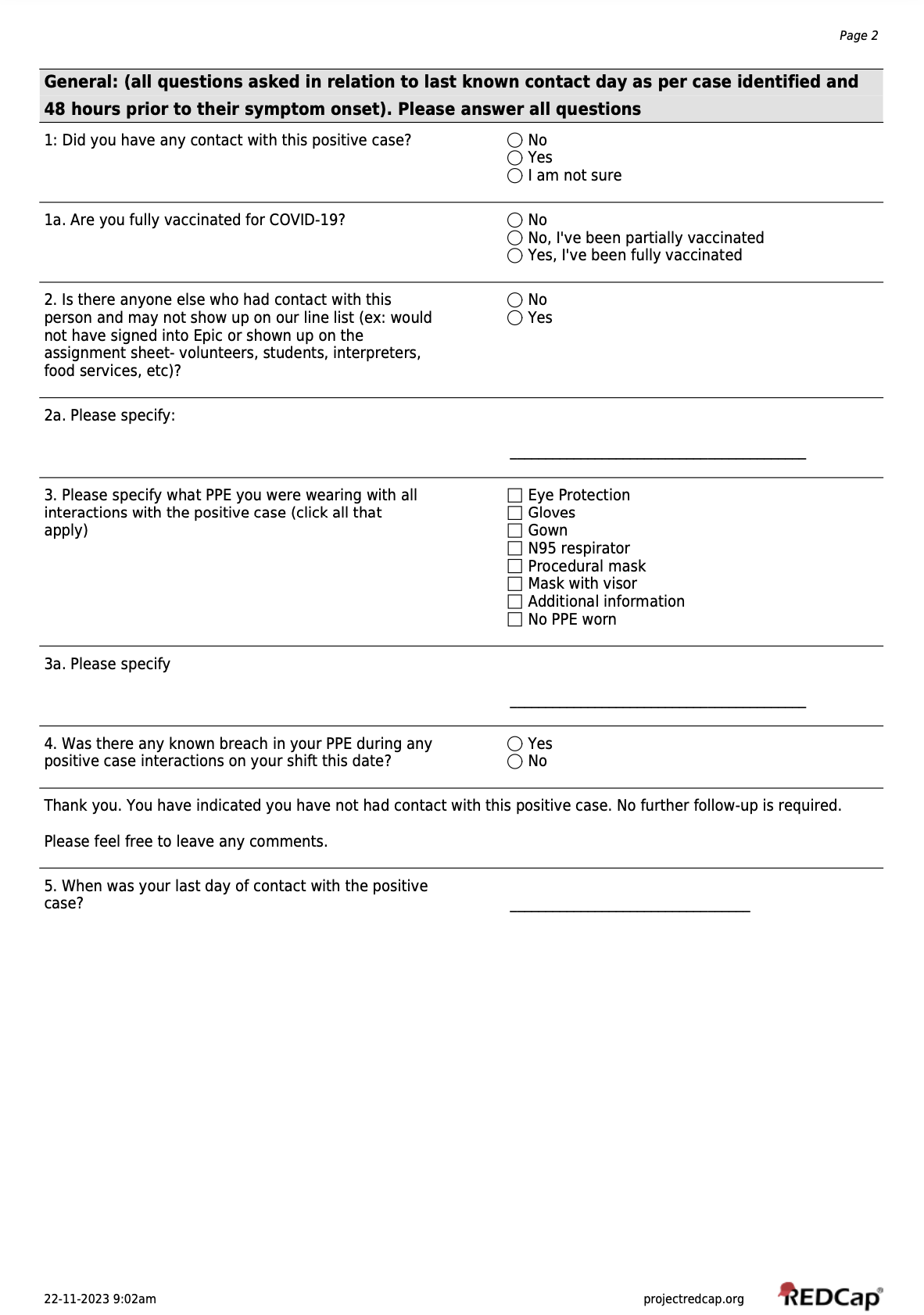


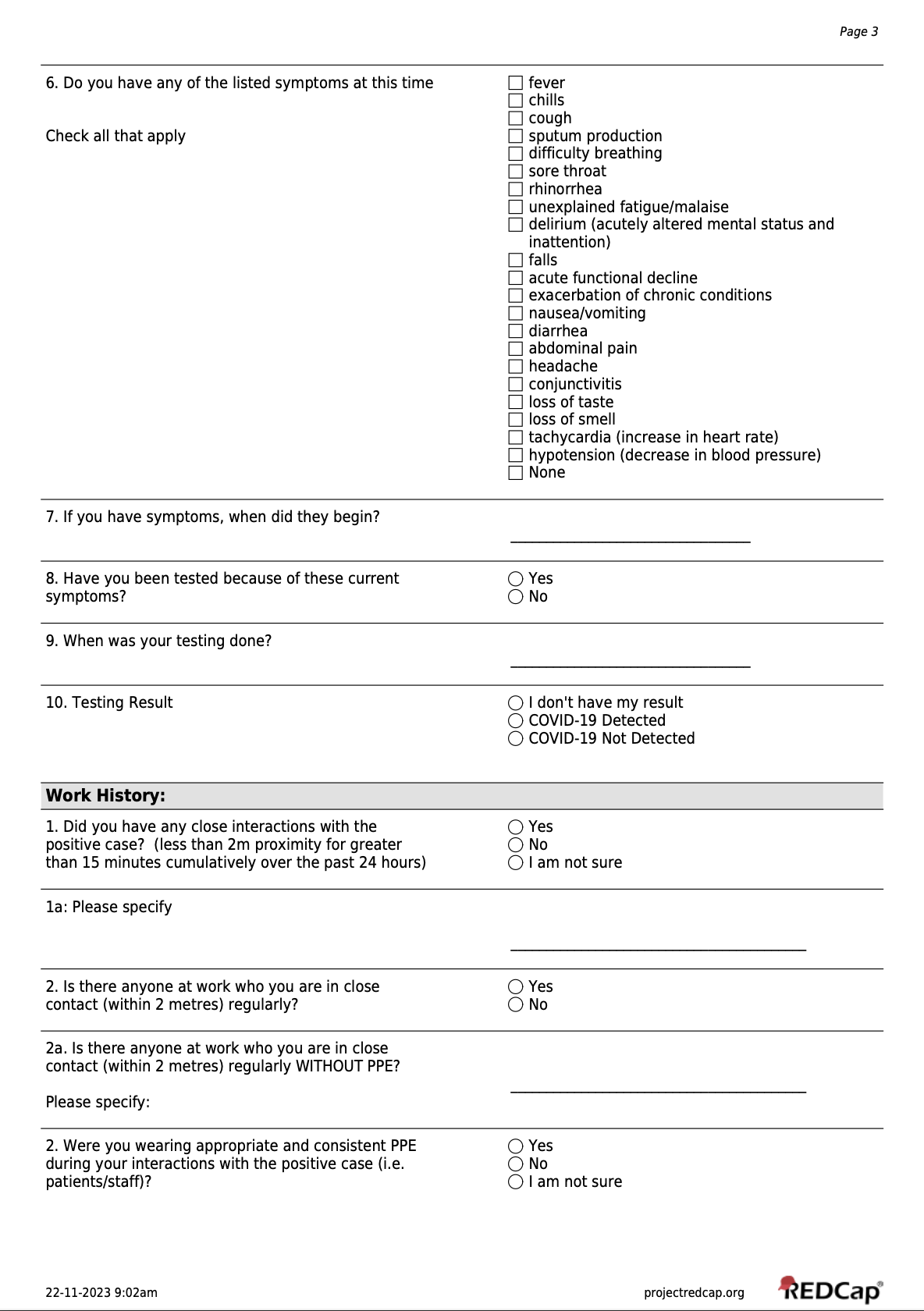


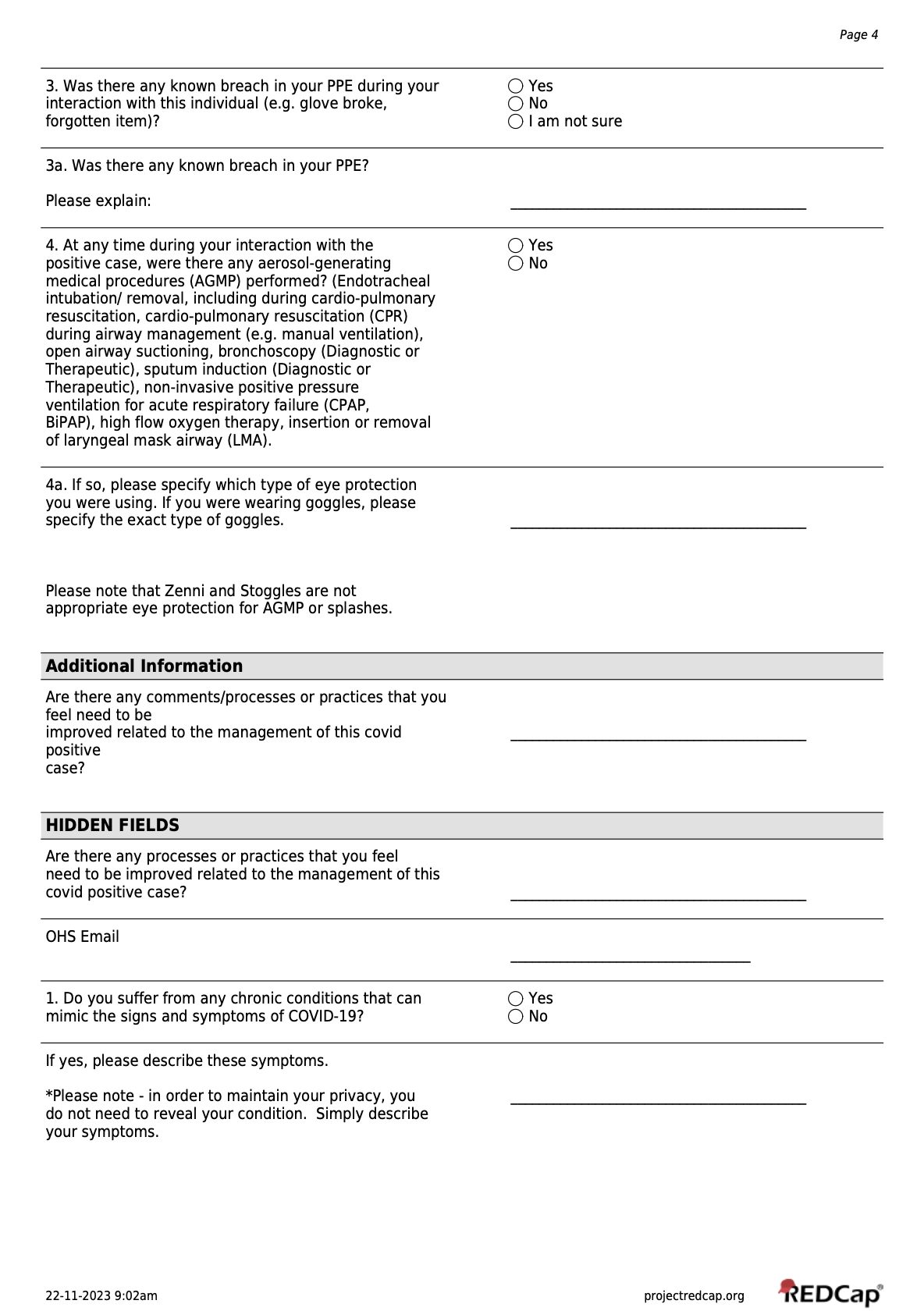


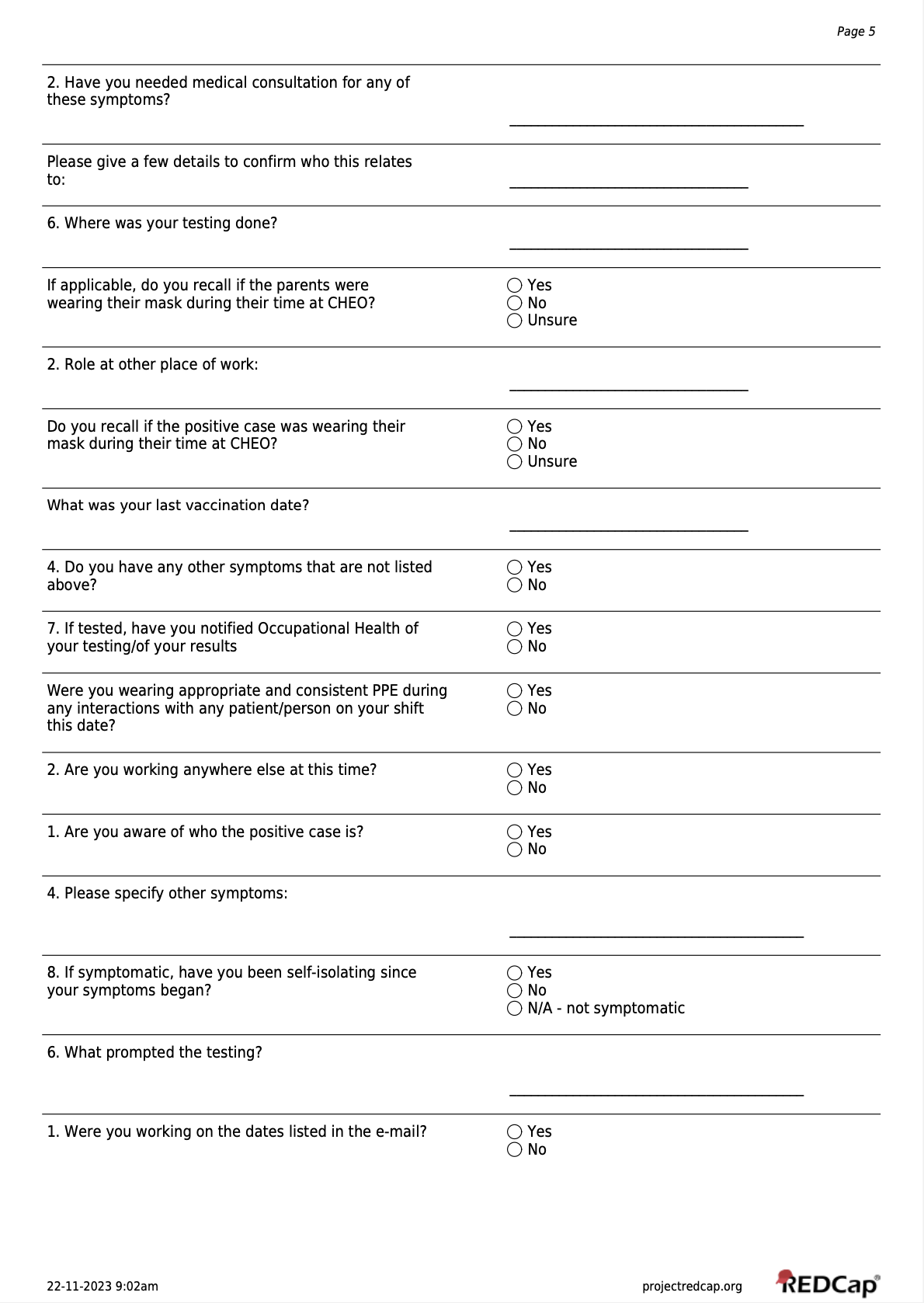


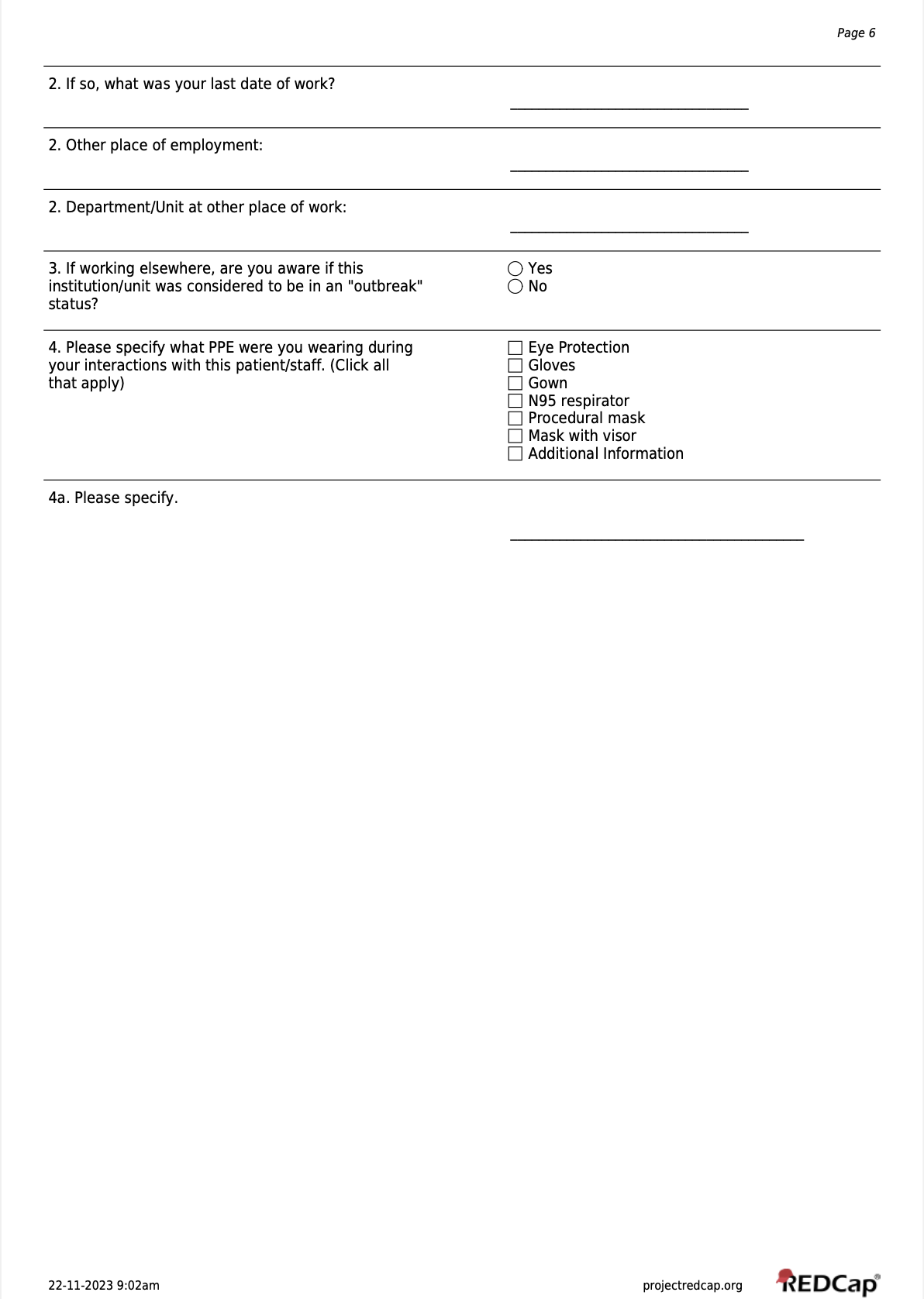


### *Interview Guide*

| INTRODUCTION:   1. To start this interview, I would like to get some brief information on your involvement at CHEO.    - 1. What is your role at the hospital?      2. How long have you been in this role?      3. Can you describe your role in the contact digital tracing initiative? |
| --- |
| REACH:   1. What was the target population identified to use the digital contact tracing tool?    1. Were there any strategies used to engage the target population to use the digital tool? 2. Who do you believe benefits from CHEO using the digital tool for contact tracing? (i.e., staff? Patients?) |
| EFFECTIVENESS:   1. From your perspective, what would you consider an effective digital contact tracing strategy to be?    1. What specific dimensions and measures would you consider if you were to assess the effectiveness of digital contact tracing? 2. Given your experience, do you consider the digital contact tracing implemented at CHEO to be effective? 3. In your opinion, what have been the main *benefits* of using the digital tool for contact tracing? 4. In your opinion, what have been the main *challenges* of using the digital tool for contact tracing? |
| ADOPTION:   1. In your opinion, do you believe the organization was successful in adopting a digital tool for contact tracing?    1. If so, what factors do you think contributed to the organization adopting the digital tool? (e.g., *facilitators*) 2. Are you aware of any barriers that may have prevented the adoption of digital tool used at CHEO? If so, what were they?    1. In your opinion, how do you think these barriers can be addressed? |
| IMPLEMENTATION:   1. What factors influenced the implementation of digital contact tracing?    1. How do you think the use of a digital tool for contact tracing has been viewed by the organization? Do you think that has changed over time? |
| MAINTENANCE:   1. Do you think that the digital contact tracing tool is sustainable in its current form?    1. From your point of view, is there anything that needs to happen to make the digital tool more sustainable? 2. Do you foresee CHEO sustaining this form of contact tracing in the future? If so, why? |
| LESSONS LEARNED AND RECOMMENDATIONS   1. Are there areas of improvement, regarding the implementation of such a tool, that you would recommend?    1. What changes could be made to the digital tool to optimize its utility? 2. Is there any additional information that you would like to share on this topic? |

*Evidence of Research Approach*

| **Research Team/Reflexivity** | |
| --- | --- |
| COREQ Guidelines [41] | Braun & Clarke [40] |
| Personal characteristics   - Which author/s conducted the interview or focus group? **Stated: under “Data Analysis”.** - What were the researcher’s credentials? **Stated: under “Data Analysis”.** - What was their occupation at the time of the study? **Stated: under “Data Analysis”.** - Was the researcher male or female? **Stated: under “Data Analysis.”** - What experience or training did the researcher have? **Stated: under “Data Analysis”.**   Relationship with participants   - Was a relationship established prior to study commencement? **Stated: under “Participant Selection and Recruitment.”** - Participant knowledge of the interviewer? **Stated: under “Participant Selection and Recruitment.”** - What did the participants know about the researcher? (e.g. personal goals, reasons for doing the research). **Stated: under “Participant Selection and Recruitment.”** - What characteristics were reported about the interviewer/facilitator? (e.g. Bias, assumptions, reasons and interests in the research topic). **Provided in Supplementary Material.** | - BO documented any ideas and reflections, decisions made, and interpretations/implicit biases throughout the research process in a journal. BO started this early in the research process (e.g., data collection) and continued to document reflexivity throughout the analysis procedures. - This research is a product of the first author’s (BO) Master’s thesis research. Her research background involves working in technology-related initiatives in various healthcare settings, ranging from increasing access to pediatric specialty care to the long-term care sector. She has a particular interest in exploring how health systems utilize and implement technologies for system improvement. - To minimize researcher bias, BO fully immersed herself in the data. This involved completing a thorough analysis, reflecting on findings, and devoting sufficient time to examining codes, emergent categories, and themes. |

| **Study Design** | |
| --- | --- |
| COREQ Guidelines [41] | Miles et al. [39] |
| Theoretical framework/Methodological orientation and Theory   - What methodological orientation was stated to underpin the study? e.g. grounded theory, discourse analysis, ethnography, phenomenology, content analysis. **Stated: under “Data Analysis.”**   Participant selection   - How were participants selected? e.g. purposive, convenience, consecutive, snowball. **Stated: under “Participant Selection and Recruitment.”** - How were participants approached? e.g. face-to-face, telephone, mail, email. **Stated: under “Participant Selection and Recruitment.”** - How many participants were in the study? **Stated: under “Results-Overview.”** - How many people refused to participate or dropped out? Reasons? **Stated: under “Results-Overview.”**   Data collection   - Were questions, prompts, guides provided by the authors? Was it pilot tested? **Stated: “Data Collection.”** - Were repeat interviews carried out? If yes, how many? **Stated: “Data Collection.”** - Did the research use audio or visual recording to collect the data? **Stated: “Data Collection.”** - Were field notes made during and/or after the interview or focus group? **Stated: “Data Analysis.”** - What was the duration of the interviews or focus group? **Stated: “Data Collection.”** - Was data saturation discussed? **Stated: “Data Collection.”** - Were transcripts returned to participants for comment and/or correction? **Stated: “Data Collection.”** | To ensure *confirmability*, steps were taken to ensure that the methods and procedures of this study were well-detailed and explicitly described. Included:   - Provided details on how the data were collected, processed, analyzed, and displayed.   To ensure *dependability*, BO actively took steps to ensure the quality and integrity of this research by having a well-defined research question, research paradigm, and study design. Included:   - BO obtained assistance from an external colleague who supported reviewing data (e.g., transcripts), the data interpretation process (e.g., coding), and process materials (e.g., memos and initial codebook). - BO actively searched for elements that nuanced the emerging data and engaged in negative case analysis. - All participants received a draft of findings for member-checking purposes. Two participants replied that they had no additional insights to be added to the findings. |

| **Analysis/Findings/Reporting** | |
| --- | --- |
| COREQ Guidelines [41] | Miles et al. [39] |
| Data analysis   - How many data coders coded the data? **Stated: “Data Analysis.”** - Description of the coding tree. **Stated: “Data Analysis.”** - Did authors provide a description of the coding tree? **Stated: “Data Analysis.”** - Were themes identified in advance or derived from the data? **Stated: “Data Analysis.”** - What software, if applicable, was used to manage the data? **Stated: “Data Analysis.”** - Did participants provide feedback on the findings? **Stated: “Data Collection.”**   Reporting   - Were participant quotations presented to illustrate the themes/findings? **Yes – throughout “Results.”** - Was each quotation identified? e.g. participant number. **Yes – throughout “Results.”** - Was there consistency between the data presented and the findings? **Yes – throughout “Results.”** - Were major themes clearly presented in the findings? **Yes – throughout “Results.”** - Is there a description of diverse cases or discussion of minor themes? **Yes – throughout “Results.”** | Providing a thorough and context-rich description of this thesis also establishes the *transferability* of this study’s findings. Includes:   - Study characteristics described in detail (e.g., setting, participants, data processes, and findings). - We specify the limitations restricting the results from being transferred to different settings and contexts. |

*Detailed Results*

Table A. Facilitators and Barriers to Reach

| Theme  (*Sub-themes*) | Key Findings | Participant Quote |
| --- | --- | --- |
| Facilitators  *Usability and Familiarity* | - Participants reported the DCT had successful reach to a broad target audience, including learners, volunteers, support staff, and medical staff. - All stakeholders highlighted the user-friendliness, convenience, and familiarity of the DCT tool’s platform influenced staff’s engagement. - End-users held similar perspectives on their personal engagement with the DCT tool. | “*For [staff] to be able to respond quickly, virtually. Instead of having to respond to a call back and play phone tag back and forth with our team.” P05, Occupational Specialist* |
| *Self-Awareness and Safety Practices* | - While most participants, including end-users, reported staff’s desire to be aware of their probable exposures and ensuring the safety of those around them in their clinical and personal environment influenced their engagement with the DCT tool, a fear of COVID-19 was also presented as a perceived factor among the other stakeholder groups. | *“I had to use the tool in order to ensure the safety of the people I was in contact with. Just to make sure there wasn't any incidental or non-incidental contact traces that put some people in high-risk factors.” P13, End-user* |
| Barriers  *Accessibility and Support* | - End-users report minimal barriers to their engagement with the DCT tool. However, all participants recognized the potential for accessibility issues (technology proficiency and access), as well as possible language concerns. | *“There could be people on certain teams that don't read English. I don't know that, but I would throw that out there.” P07, Implementor* |
| *Concerns of Risk-Assessment* | - Stakeholders highlighted that a barrier to staff’s engagement may have existed in the implications and perceived negative consequences for completing the risk-assessment, such as temporary work status. | *“Some people might not have understood even, why? Why would you? Why are you asking all these questions about me? People could have been suspicious to say, ‘Well are you asking me these questions because you want to see if I did something wrong? You want me to say that I didn't wash my hands, or I didn't wear my PPE when I was in proximity? Are you trying to get me in trouble?’” P07, Implementor* |

Table B. Summary of Effectiveness

| Theme  (*Sub-themes*) | Key Findings | Participant Quote |
| --- | --- | --- |
| Positive Impacts  *Agile Response to COVID-19* | - Participants reported benefits, such as DCT scalability, efficiency, and flexibility in meeting the hospital’s needs during COVID-19. | *“For contact tracing, we were able to go back and determine whether there may have been a potential exposure related to surveys that were filled out and kind of map everything together. If we were trying to figure out if there was a transmission within the hospital.” P11, Occupational Specialist* |
| *Informed Decision Making* | - Positive impacts of the DCT tool as perceived by all stakeholder groups also stemmed from improved COVID-19 knowledge and decision-making. - At the individual level, participants acknowledged that staffs’ acquired awareness gained through the DCT tool facilitated improved safety for patients and their families, staff, and the broader community during the pandemic. - At organizational level, implementors and occupational specialists highlighted that access to real-time data captured by the DCT tool informed evidence-based infection control decision making. | *“So, then going through the questionnaire kind of made you realize, ‘Oh, I'm okay. I'm completely okay. Or maybe I need to sit this one out.’” P17, End-user*  *“If you're working in neonates (…) working in self-isolation, you have a greater understanding and heightened awareness. That, ‘I've been in contact with COVID, I need to monitor my symptoms for 10 days.’ So, it's a heightened awareness of keeping your PPE proper. You should always keep it proper, and you should never breach it. But it keeps you that more aware. Because now you could impact that little 24-weeker.” P15, Occupational Specialist* |
| Challenges and Unintended Outcomes  *Technology Limitations* | - Stakeholders identified various issues related to data quality captured by the DCT, alongside the general contact tracing process. This included recall limitations, manual errors, and the DCT’s inability to capture nuanced information - Stakeholders highlighted the issues with the platform utilized for DCT, including server capacity issues. | *“… Because there is recognition that you could be, face-to-face with someone, and they're not masked. Versus face-to-face with a mask, versus at the back of the room, or at the foot of the bed, or talking to them at the door. So, that nuance is lost with this questionnaire.” P09, Occupational Specialist* |
| *Staffing Impacts* | - Negative implications of the DCT tool were infrequently mentioned; however, stakeholder groups did consider the impacts of contact tracing, the delivery of the DCT tool, and other system level factors on staffing. | *“Let's say an office worker was a high-risk exposure. Well, that's not really affecting the patients. But the frontline staff, it really affected [the patients], because they had to be off and all that.” P10, Occupational Specialist* |
| Indicators of Effectiveness | See Table 3 for detailed information on indicators. | |

Table C. Facilitators and Barriers of Adoption

| Theme  (*Sub-themes*) | Key Findings | Participant Quote |
| --- | --- | --- |
| Facilitators  *Safety-Focused Culture* | - Adoption was facilitated by a strong collective commitment to staff safety during the pandemic, including financial incentives for staff who contracted COVID-19. | *“I guess maybe through internal communication. They'd always have these COVID updates on a regular basis, to keep everyone on board and on the same page. What was going on and update the staff on the changes. And in there, they would talk about the contact tracing and encouraged staff to fill out our tool that we had.” P19, Occupational Specialist* |
| *Personalized Guidance* | - Engagement of trusted and well-established nurses acting behind the technology influenced staff receptivity to DCT. | *“[The CST] were a very trusted group of people. People were really counting on those nurses to keep them safe in the hospital.” P12, Implementor* |
| Barriers  *Misalignment with Frontline Practices* | - Barriers to adoption included the DCT tool not aligning well with standard practices of frontline HCWs, particularly in clinical areas. | *“You don't necessarily get the responses from doctors and nurses, but you'll get them from others. No pharmacy, no doctors, and most likely no nurses. Then if you have a social worker, all those [allied health professionals], I feel like they respond more than everybody else.” P08, Occupational Specialist* |
| *Sustaining Participation* | - HCWs experiencing high COVID-19 fatigue and evolving perspectives impacted sustained adoption of the DCT tool. | *“There are roles in the hospital that just aren't great at using email and it was very much an email-based system. If you're in environmental services and things like that, you may not actually be checking your email that often. In which case, the whole thing falls apart because it's triggered by email.” P12, Implementor* |

Table D. Summary of Implementation

| Theme  (*Sub-themes*) | Key Findings | Participant Quote |
| --- | --- | --- |
| Factors Influencing Implementation  *Maintaining Compliance* | - Implementors expressed the necessity of contact tracing and disease surveillance mandated by governing bodies (Ontario Ministry of Health and Long-term Care) | *“… We were regularly getting dictums from the Ministry of Health saying, ‘thou shalt screen all patients before they come in, screen all staff before they come in, report all exposures, report all positive cases.” P12, Implementor*  *“I think if public health didn't do it, I don't know if we would have started to do it. We sort of follow what they do, they come out with their guidance, and we just follow what the guidance says.” P10, Occupational Specialist* |
| *Maximizing Efficiency* | - Implementors and occupational specialists recognized the need for systematic contact tracing and opted to utilize technology to streamline the process and ensure data accuracy. - The pre-existing use of REDCap for other hospital mechanisms (e.g. entrance screening). | *“I know that there was a lot of pressure on the system. That would have resulted in huge delays, and in contact tracing that would have rendered it quite meaningless if there hadn't been a way to sort of speed it up. … I would [say] that from a leveraging technology standpoint, the ability to do it with less people, and quickly, and to get meaningful data that was standardized, to get all that back in real-time. I think would have weighed heavily on the decision to go ahead and implement it.” P07, Implementor* |
| *Fostering Trust and Confidence* | - Implementors recognized building trust among staff was crucial for implementing the DCT tool, as it aimed to ensure safety in the hospital environment amidst the uncertainties of COVID-19. | *“The number one internal thing. Well, internal and external, people were scared, right? People were really, really scared at the beginning of COVID. We didn't really know what this thing was, we didn't know how it was transmitted, and we didn't know how sick people were going to get. People were scared to come to work. It was really, really important to build trust among our staff, that we were doing all we could to protect them. So, that they could feel safe coming to work. That was one of the primary sorts of internal reasons for doing this, is we knew sick people would be coming into the hospital.” P12, Implementor* |
